# Evaluation of the relationship between HbA1c level and retina choroidal thickness in patients with gestational diabetes mellitus

**DOI:** 10.5935/0004-2749.20220045

**Published:** 2025-08-21

**Authors:** Bekir Kahveci, Yıldız Dilbade Ekinci

**Affiliations:** 1 Department of Obstetrics and Gynecology, Cukurova University, Adana, Turkey; 2 Department of Ophthalmology, Health Sciences University, Gazi Yasargil Research and Training Hospital, Diyarbakir, Turkey

**Keywords:** Gestational diabetes mellitus, HbA1c, Central macular thickness, Central choroid thickness, Optical coherence tomography, Diabetes mellitus gestacional, HbA1c, Espessura macular central, Espessura da coroide central, Tomografia de coerência óptica

## Abstract

**Purpose:**

To investigate the effect of hemoglobin A1c level on central macular thickness and central, nasal, and temporal choroidal thickness in patients with gestational diabetes mellitus.

**Methods:**

This retrospective study included 41 patients who had been diagnosed with gestational diabetes mellitus and undergone a 75-g oral glucose tolerance test between 24 and 28 weeks of gestation. They were divided into two groups based on their hemoglobin A1c level (group 1: hemoglobin A1c <6.0% and group 2: hemoglobin A1c ≥6.0%). All patients underwent a complete ophthalmologic examination. The central macular thickness and central, nasal, and temporal choroidal thickness were measured using optical coherence tomography. **Results: O**f the 3,016 pregnant women screened, 7.5% (n=228) were diagnosed with gestational diabetes mellitus during the study period and 41 of these patients were included in the study. Group 1 comprised 48 eyes from 24 patients and Group 2 consisted of 34 eyes of 17 patients. The average body mass index values were 30.8 ± 3.3 and 35.1 ± 9.0, respectively (p=0.002). The insulin use rates were 29.2% and 76.5%, respectively (*p*=0.000). Mean central macular thickness values were 250.8 ± 14.3 µm and 260.9 ± 18.1 µm, respectively, and the difference was significant (p=0.008).

**Conclusions:**

Although the body mass index and central macular thickness values were significantly higher in Group 2, there was no difference in the central, nasal, and temporal choroidal thickness between the two groups.

## Abstract (pt)

**Objetivos:**

Investigar o efeito do nível de hemoglobina A1c (HbA1c) na espessura macular central e na espessura da coróide central, nasal e temporal em pacientes com diabetes mellitus gestacional.

**Métodos:**

Este estudo retrospectivo incluiu 82 olhos de 41 pacientes diagnosticadas com diabetes mellitus gestacional, as quais fizeram um teste de tolerância oral à glicose de 75 g entre 24 e 28 semanas de gestação. As pacientes foram divididas em dois grupos de acordo com o nível de hemoglobina A1c (hemoglobina A1c <6,0% e hemoglobina A1c ≥6,0%). Todas as pacientes foram submetidas a exame oftalmológico completo e, a espessura macular central, a espessura central, nasal e temporal da coroide foram mensuradas por tomografia de coerência óptica.

**Resultados:**

Durante o período do estudo, das 3.016 gestantes triadas, 7,5% (n=228) foram diagnosticadas com diabetes mellitus gestacional. Destas, 41 pacientes foram analisadas de acordo com os critérios do estudo. Houve 48 olhos de 24 pacientes no primeiro grupo com hemoglobina A1c <6,0% e 34 olhos de 17 pacientes no segundo grupo com hemoglobina A1c ≥6,0%. Os valores médios do índice de massa corporal foram de 30,8 ± 3,3 e 35,1 ± 9,0, respectivamente (*p*=0,002). As taxas referentes ao uso de insulina foram de 29,2% e 76,5%, respectivamente (*p*=0,000). Os valores médios da espessura macular central foram medidos em 250,8 ± 14,3 µm e 260,9 ± 18,1 µm, respectivamente e a diferença foi significativa entre os dois grupos (*p*=0,008).

**Conclusões:**

Embora os valores do índice de massa corporal e da espessura macular central tenham sido significativamente maiores no Grupo 2 com hemoglobina A1c alta, não houve diferenças nas medidas de espessura coroidal central, nasal e temporal entre os dois grupos.

## INTRODUCTION

Gestational diabetes mellitus (GDM) occurs in women with no history of high blood sugar before pregnancy^(1)^. The main risk factors for GDM are obesity, malnutrition, history of GDM or diabetes, advanced maternal age, positive family history, and genetic predisposition^(2)^. The worldwide prevalence of GDM is 6%^(3)^. Insulin sensitivity increases in the first week of a healthy pregnancy. However, as the gestational age progresses, insulin resistance develops due to the maternal and placental hormones, causing a slight increase in the blood sugar level. When there is no corresponding increase in the insulin level to counter the elevated blood sugar level, it leads to glucose intolerance and GDM in susceptible mothers^(4)^. Although the blood sugar is constantly monitored after delivery, there is an increased risk of type 2 diabetes mellitus (T2DM), hypertension, and cardiovascular disease in mothers diagnosed with GDM^(5,6)^.

Ophthalmologic examination of patients with GDM is usually ignored as retinopathy is not common in this group. However, the literature contains studies reporting complications that may cause serious ocular morbidities such as retinopathy, glaucoma, and cataracts in these patients^(7-9)^. Optical coherence tomography (OCT) studies have revealed that the choroid thickness is affected in patients with GDM^(10,11)^. Also, as some patients diagnosed with GDM might have undiagnosed T2DM, retinal scans have been recommended for this patient group^(12)^.

Hemoglobin A1c (HbA1c) test is used to evaluate glycemic control in the previous 2-3 months of pregnancy in diabetic patients^(13)^. However, while one study stated that HbA1c level can be used for GDM diagnosis in pregnant women instead of an oral glucose tolerance test (OGTT)^(14)^, another study demonstrated that the use of this parameter is not convenient for patients with HbA1c level <4.5%, considering that GDM can be diagnosed without OGTT in pregnant women with HbA1c levels >5.8%^(15)^. Although there is a relationship between increased HbA1c level and diabetic retinopathy in diabetic patients^(16)^, it has not been investigated in patients with GDM.

In this study, we analyzed the effect of HbA1c level on central macular thickness (CMT) and central, nasal, and temporal choroidal thickness (CCT, NCT, and TCT, respectively) in patients diagnosed with GDM.

## METHODS

This study was conducted at the Health Sciences University, Gazi Yaşargil Training and Research Hospital, in accordance with the Helsinki protocol after the approval of the ethics committee (Number: 28.06.2019/302). Patients who had a history of diabetes, other endocrinological diseases, or drug use history that could affect the blood glucose level; cardiovascular or renal disease; had undergone an intraocular surgery; had any ocular disease; or whose spherical equivalent was higher than ± 1, and the best-corrected visual acuity was below 10/10, were excluded from the study. A total of 41 patients diagnosed with GDM and fulfilling the inclusion criteria were included in this study.

As per the International Association of Diabetes and Pregnancy Study Group’s guidelines^(17)^, GDM was diagnosed using a 75-g OGTT in patients at 24-28 weeks of gestation. Fasting blood sugar was measured in these patients after at least 8 hours of fasting, after which they were given 75 g of oral glucose. The blood glucose level was measured again after the first and second hour of oral glucose administration. Patients with initial fasting blood sugar of 92 mg/dL, first-hour blood sugar of 180 mg/dL, or second-hour blood sugar of 153 mg/dL were diagnosed with GDM.

Information regarding the patients’ age, number of pregnancies and live births, gestational age, body mass index (BMI), HbA1c levels, insulin use, and diet was recorded. They were divided into two groups according to their HbA1c level (group 1: HbA1c <6.0%; group 2: HbA1c ≥6.0%).

The patients’ eye examinations were also perfor med in our clinic, which included best-corrected visual acuity assessment based on the Shellen chart, biomicroscopy, intraocular pressure measurement using Goldmann Applanation Tonometry, and dilated fundus examination. Retinal and choroidal thickness were measured with spectral domain OCT (Heidelberg Engineering, Dossenheim, Germany) by an experienced technician. Simultaneously, the CMT was derived from the values obtained from OCT, and the choroidal thickness was mea sured using the OCT’s enhanced deep imaging mode. The choroidal thickness was calculated manuallye by measuring the distance between the outer boundary of the retinal pigment epithelium and the inner edge of the sclera. The CMT, CCT, NCT, and TCT at a distance of 1 mm from the fovea were compared between the two groups.

### Statistical analysis

The variables used for descriptive statistics included the average, standard deviation, median, frequency, and ratio values. The Kolmogorov-Smirnov test was used to analyze the distribution of variables. Independent-samples *t*-test and Mann-Whitney U-test were used in the analysis of quantitative independent variables, whereas Chi-square test was used for qualitative independent variables. The SPSS 26.0 software was used for the statistical analysis.

## RESULTS

**Figure 1:**
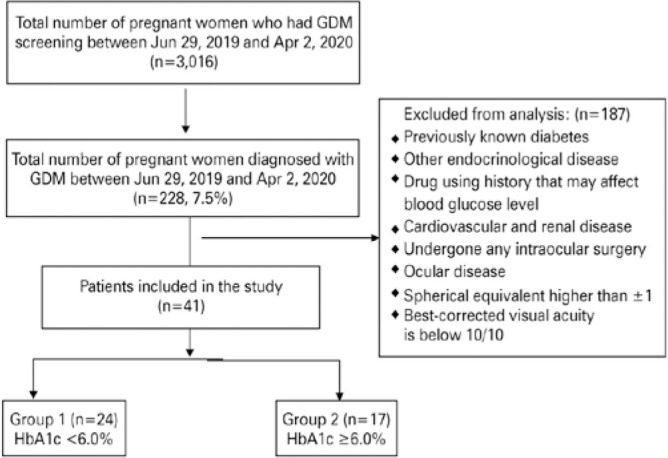
Study flowchart.

A total of 3,016 pregnant women were screened during the study period and 228 (7.5%) of them were diagnosed with GDM. Out of those 228 patients, 41 women whose gestational age was between 24 and 28 weeks were included in this study. Group 1 (HbA1c <6.0%) had 24 patients (48 eyes), and group 2 (HbA1c ≥6.0%) had 17 patients (34 eyes). The distribution of the patients diagnosed with GDM during the study period is summarized in figure 1. The mean p or average values of the different parameters of the patients in groups 1 and 2, respectively p, measured during the study were as follows: age: 32.5 ± 6.3 years and 34.7 ± 5.6 (p=0.089); BMI: 30.8 ± 3.3 and 35.1 ± 9.0 (p=0.002); and gestational age during the ophthalmologic evaluation: 30.9 ± 3.2 weeks and 31.9 ± 3 weeks (p=0.246). The demographic data of both the groups is summarized in table 1.

**Table 1:** Demographic data of the groups

	Group 1 (n=24) HbA1c <6.0%	Group 2 (n=17) HbA1c ≥6.0%	p-value
Age^a^	32.5 ± 6.3	34.7 ± 5.6	0.089
Gravida^a^	5.25 ± 2.7	4.70 ± 2.8	0.078
Parity^a^	3.1 ± 2.1	2.5 ± 1.9	0.061
Gestational age^a^	30.5 ± 3.2	31.9 ± 3.0	0.246
BMI (kg/m^2^)^a^	30.8 ± 3.3	35.1 ± 9.0	0.002^*^
Insulin use^b^	7 (29.2)	13 (76.5)	0.000^*^

BMI= Body Mass Index; HbA1c= Hemoglobin A1c;

a = Data are given as mean ± standart deviation;

b = Data ara presented as number (percentage); ^*^P≤0.05= statistically significant.

GDM= Gestational diabetes mellitus; HbA1c= Hemoglobin A1c.

The mean HbA1c level was 5.4 ± 0.3% in group 1 and 7.0 ± 1.5% in group 2. During the treatment for GDM, 15 (62.5%) patients in group 1 and 11 (64.7%) patients in group 2 followed a specific diet. The insulin use rates were 7 (29.2%) and 13 (76.5%), respectively (p=0.000).

**Figure 2:**
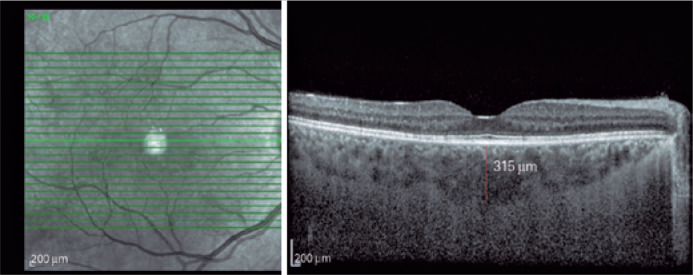
Central choroidal thickness measurement of a GDM patient whose HbA1c level was 5.9%.

The CMT values for groups 1 and 2 were 250.8 ± 14.3 µm and 260.9 ± 18.1 µm, respectively, and the diffe rence was statistically significant p (p=0.006). table 2 shows a comparison of the CMT, CCT, NCT, and TCT values between the 2 groups. There was no statistically significant difference between CCT (Figure 2), NCT, and TCT quantification between the groups (p>0.05).

**Table 2:** Measurement of the central macular thickness and central, nasal, and temporal choroid thicknesses between the groups.^a^

	Group 1 (n=24) HbA1c <6.0%	Group 2 (n=17) HbA1c ≥6.0%	p-value
CMT	250.8 ± 14.3	260.9 ± 18.1	**0.006** ^*^
CCT	332.0 ± 59.4	317.7 ± 19.3	0.451
NCT	318.0 ± 23.5	302.2 ± 14.4	0.675
TCT	341 ± 28.8	326.3 ± 21.1	0.498

CMT= Central macular thickness; CCT, Central choroidal thickness; NCT= Nasal choroidal thickness; TCT= Temporal choroidal thickness; HbA1c= Hemoglobin A1c;

a = Data are given as mean ± standart deviation (Micrometer); ^*^P≤0.05= statistically significant.

## DISCUSSION

DM is a chronic disease with long-term ocular complications such as retinopathy, cataracts, glaucoma, and optic neuropathy^(18-20)^. Diabetic retinopathy is the most common complication of DM affecting the eyes^(18)^, which could lead to loss of vision if left untreated. Its severity is closely related to elevated HbA1c level, duration of diabetes, comorbidities such as hyperlipidemia and hypertension, puberty, and pregnancy^(21)^. The choroidal and retinal vascular structures in diabetic patients have been reported to be affected even without developing retinopathy, based on the OCT studies in recent years^(10,22)^.

The risk of retinopathy progression was noted to be significantly high in pregnant diabetic women^(23)^. Some studies have reported that there were no retinopathy findings in patients with GDM, similar to our study^(7,10,11)^. However, some other studies have observed proliferative retinopathy, retinal arteriolar narrowing, decreased retinal arteriolar fractal dimension, and larger retinal arteriolar branching angle in GDM patients^(24,25)^. It is assu med that temporary hyperglycemia from GDM leads to arteriolar vasoconstriction, causing arteriolar narrowing^(25)^. In addition, scholars have argued that the retinal vascular morphological abnormalities in GDM patients could be a result of hypoxia due to hyperglycemia^(25)^. It is not yet known whether these vascular changes have prognostic significance for the development of T2DM in future. However, close monitoring of this patient group may promote early T2DM diagnosis and help in the prevention of severe ocular and systemic complications associated with it.

As obesity is implicated as a predisposing factor for the development of GDM, weight loss and exercise can help control this disease^(26)^. Furthermore, in some studies, obesity has been identified as a risk factor for the development of postpartum DM in addition to GDM^(27)^. Another critical risk factor for postpartum DM is an elevated HbA1c level in the third trimester of pregnancy^(28)^. The use of insulin for glycemic control during pregnancy has also been considered as a risk factor for T2DM in the postpartum period^(29)^. In our study, considering the increased BMI and high rate of insulin use in group 2, we felt that these patients are more likely to develop diabetes in future and that they should be followed up closely in the postpartum period.

The retina is the most frequently affected ocular tissue in diabetic patients with hyperglycemia. Diabetic retinopathy is caused by an increase in the vascular endothelial growth factor due to poor diabetes control, leading to an increase in the macular thickness, as well as the development of macular edema^(30)^. Although there are not many studies in the literature that have evaluated CMT in patients with GDM, one study reported the CMT to be 252 µm in these patients. Analysis of the results revealed that the macular thickness decreased in patients with GDM compared to nonpregnant women^(11)^. In our study, the CMT was higher in patients with poor glycemic control compared to those with a good control over their blood sugar levels. Our study also demonstrated that poorly controlled DM increases the retinal thickness.

With the help of the enhanced deep imaging mode of the OCT, the choroid layer has been shown to be affected in diabetic patients^(10,11,22,30,31)^. It has been established that a decrease in the choroidal thickness in diabetic patients is associated with the worsening of retinopathy^(22)^. This could be attributed to the thinning of the choriocapillaris layer, causing hypoxia especially in the outer retinal folds, and increased vascular endothelial growth factor levels related to the hypoxia. A very recent study demonstrated that CT increased during the early stages of retinopathy but decreased in the advanced stages. It also showed that there was no correlation between macular CT and HbA1c^(31)^.

A study that compared the choroidal thickness of patients with type 1 DM (T1DM), T2DM, and GDM reported that CCT was significantly lower in patients with T1DM, but there was no difference in GDM and T2DM patients^(10)^. Another study also found no difference in the choroidal thickness between GDM patients and healthy pregnant women^(11)^. In our study, although the CCT, NCT, and TCT showed thinning in group 2 with poorly controlled diabetes compared to group 1, there was no statistically significant difference. The CT is increased in healthy pregnant women as a result of hemodynamic changes and increase in the ocular blood flow during pregnancy^(32-34)^. The choroidal thinning in patients with T1DM and T2DM, and the absence of changes in patients with GDM can be explained by this increased ocular blood flow during pregnancy, which causes an increase in the choroidal thickness and masks choroidal thinning.

In conclusion, considering the increased CMT measurements in GDM patients with elevated HbA1c levels along with elevated BMI and high insulin use rates, these patients may be at an increased risk of developing T2DM in future. However, prospective studies in with a larger study group are recommended to fully define these parameters as risk factors for T2DM development.
